# Sleep Disturbance and Burnout in Emergency Department Health Care Workers

**DOI:** 10.1001/jamanetworkopen.2023.41910

**Published:** 2023-11-03

**Authors:** Ari Shechter, Tsion Firew, Maody Miranda, Nakesha Fray, Allison A. Norful, Alvis Gonzalez, Bernard P. Chang

**Affiliations:** 1 Center for Behavioral Cardiovascular Health, Department of Medicine, Columbia University Irving Medical Center, New York, New York; 2 Department of Emergency Medicine, Columbia University Irving Medical Center, New York, New York; 3 Columbia University School of Nursing, New York, New York

## Abstract

This cross-sectional study examines the association of sleep disturbances with burnout among emergency medicine health care workers.

## Introduction

Health care worker (HCW) burnout is endemic, with rates as high as 60%.^1^ In addition to job demand–related work factors (eg, staffing levels and administrative tasks),^2^ person-level behavioral and psychosocial factors may affect burnout. For example, sleep disturbance is linked to fatigue, enhanced reactivity to negative emotions, reduced reactivity to positive stimuli, and lower self-esteem, helping behavior, and empathy toward others.^3,4^ We examined the association of sleep with burnout in emergency medicine HCWs.

## Methods

This was a cross-sectional analysis of a convenience sample of HCWs from emergency departments in a large urban medical center. Participants completed a single online-based questionnaire on demographics, sleep, and burnout. Data were collected between November 2020 and January 2022. Study procedures were approved by the Columbia University Medical Center institutional review board. All participants provided informed consent electronically. This report followed the STROBE reporting guideline. Additional methodological details are provided in the eAppendix in Supplement 1. Sleep quality and insomnia symptoms were assessed with the Pittsburgh Sleep Quality Index (PSQI) and Insomnia Severity Index (ISI), respectively. Burnout symptoms were assessed with the Abbreviated Maslach Burnout Inventory–9, which includes 3 burnout subscales: emotional exhaustion (EE), depersonalization (DP), and reduced personal accomplishment (PA). Partial correlations were calculated for the association between sleep and burnout (continuous scores). Binary logistic regressions were computed for odds ratios (ORs) on the association of the presence of poor sleep quality (PSQI score >5) and insomnia symptoms (ISI score ≥8) with the presence of burnout symptoms (ie, PA score <9, DP score >6, and EE score >9). Analyses were adjusted for age, sex, race and ethnicity (self-report), and depression symptoms, all of which are factors known to be associated with sleep.

## Results

The sample included 126 participants (mean [SD] age, 40.9 [9.5] years; 79 women [62.7%]). Demographic details and PSQI, ISI, PA, DP, and EE scores are shown in (Table 1). PSQI score was positively associated with DP (*r* = 0.27) and EE (*r* = 0.23), but not PA (*r* = −0.12). ISI score was positively associated with DP (*r* = 0.26) and EE (*r* = 0.30), but not PA (*r* = −0.04). Poor sleep (vs good) was associated with the presence of elevated EE (adjusted OR, 2.45; 95% CI, 1.02-5.89). Insomnia symptoms (vs not) were associated with the presence of elevated EE (adjusted OR, 2.56; 95% CI, 1.11-5.93). Poor sleep and insomnia symptoms (vs absence) were not significantly associated with elevated DP or reduced PA (Table 2).

**Table 1.** Demographics, Sleep, and Burnout Characteristics of the Sample and by Clinical Role

Characteristic	Participants, No. (%)					
	Full sample (N = 126)	Attending physician (n = 56)	Registered nurse (n = 29)	Resident or fellow (n = 9)	Advanced practice practitioner (n = 15)	Allied health professional (n = 17)
Age, mean (SD), y	40.9 (9.5)	42.5 (9.5)	39.7 (9.0)	33.1 (4.6)	35.5 (8.4)	46.7 (9.3)
Sex						
Female	79 (62.7)	32 (57.1)	21 (72.4)	3 (33.3)	12 (80.0)	11 (64.7)
Male	47 (37.3)	24 (42.9)	8 (27.6)	6 (66.7)	3 (20.0)	6 (35.3)
Race						
American Indian or Native American	2 (1.6)	1 (1.8)	1 (3.4)	0	0	0
Asian	26 (20.6)	14 (25.0)	8 (27.6)	1 (11.1)	1 (6.7)	2 (11.8)
Black	13 (10.3)	3 (5.4)	2 (6.9)	1 (11.1)	1 (6.7)	6 (35.3)
White	66 (52.4)	32 (57.1)	13 (44.8)	7 (77.8)	11 (73.3)	3 (17.6)
Other or >1 race^a^	10 (7.9)	3 (5.4)	3 (10.3)	0	0	4 (23.5)
Declined to respond	9 (7.1)	3 (5.4)	2 (6.9)	0	2 (13.3)	2 (11.8)
Ethnicity, Hispanic or Latino	23 (18.3)	7 (12.5)	5 (17.2)	2 (22.2)	2 (13.3)	7 (41.2)
Sleep						
PSQI score, mean (SD)	7.2 (3.1)	6.6 (2.5)	8.2 (3.9)	7.4 (2.8)	6.3 (3.4)	7.6 (3.0)
Presence of poor sleep quality (PSQI score >5)	80 (63.5)	32 (57.1)	23 (79.3)	7 (77.8)	7 (46.7)	11 (64.7)
ISI score, mean (SD)	8.4 (4.7)	8.6 (4.7)	9.3 (5.3)	9.2 (5.1)	7.1 (4.8)	6.9 (4.2)
Presence of insomnia symptoms (ISI score ≥8)	74 (58.7)	36 (64.3)	20 (69.0)	4 (44.4)	8 (53.3)	6 (35.3)
Burnout						
PA score, mean (SD)	12.2 (3.8)	12.3 (3.8)	12.6 (3.2)	13.2 (2.6)	13.1 (3.2)	9.7 (4.6)
Low PA (score <9)	22 (17.5)	11 (19.6)	4 (13.8)	0	1 (6.7)	6 (35.3)
DP score, mean (SD)	4.7 (4.1)	4.5 (4.1)	5.1 (4.4)	6.2 (5.5)	4.3 (3.8)	4.1 (4.2)
High DP (score >6)	35 (27.8)	17 (30.4)	9 (31.0)	3 (33.3)	2 (13.3)	4 (23.5)
EE score, mean (SD)	8.4 (4.5)	7.9 (4.5)	9.2 (4.0)	8.9 (5.8)	9.3 (3.8)	7.3 (5.5)
High EE (score >9)	54 (42.9)	22 (39.3)	14 (48.3)	3 (33.3)	8 (53.3)	7 (41.2)

Abbreviations: DP, depersonalization; EE, emotional exhaustion; ISI, Insomnia Severity Index; PA, personal accomplishment; PSQI, Pittsburgh Sleep Quality Index.

^a^ Other and more than 1 race was a broad category without specific subcategories.

**Table 2.** Association of Poor Sleep Quality and Insomnia Symptoms With Burnout Components^a^

Variable	Adjusted OR (95% CI)^b^
Reduced personal accomplishment	
Poor sleep quality	2.81 (0.79-9.97)
Insomnia	0.60 (0.20-1.75)
Elevated depersonalization	
Poor sleep quality	2.48 (0.92-6.71)
Insomnia	1.99 (0.78-5.07)
Elevated emotional exhaustion	
Poor sleep quality	2.45 (1.02-5.89)^c^
Insomnia	2.56 (1.11-5.93)^c^

Abbreviation: OR, odds ratio.

^a^ Poor sleep quality is defined as a Pittsburgh Sleep Quality Index score greater than 5. Insomnia is defined as a Insomnia Severity Index greater than or equal to 8.

^b^ Data were analyzed with binary logistic regression and adjusted for age, sex, race and ethnicity, and depression symptoms.

^c^ *P* < .05.

## Discussion

Sleep disturbance is common among emergency medicine HCWs. Most participants in this cross-sectional study reported poor sleep and insomnia symptoms, both of which were associated with burnout. Study limitations include the small sample size, a convenience sample limiting external generalizability and internal validity, and a cross-sectional analysis with a single assessment that precludes conclusions about causal relationships. Although not assessed here, the sleep-burnout relationship is likely bidirectional. HCWs’ poor sleep may also be associated with factors unrelated to occupational demands (eg, personal stressors and family obligations). Other unaccounted for external factors (eg, seasonal variations in patient volume and acuity) could have impacted findings. Strengths include the use of 2 validated tools to assess sleep quality and insomnia and a multidimensional assessment of burnout. The current findings add to evidence linking sleep to burnout.^5^ Future work should examine whether individual-level sleep interventions moderate the impact of organizational and system-level approaches (eg, increased staffing, reduced clerical burden, and circadian-based scheduling) on burnout.^6^

## Notes

Supplement 1. **eAppendix.** Supplemental Methods
eReferences

Supplement 2. Data Sharing Statement

## References

[1] Linzer M, Jin JO, Shah P, . Trends in clinician burnout with associated mitigating and aggravating factors during the COVID-19 pandemic. JAMA Health Forum. 2022;3(11):e224163. doi:10.1001/jamahealthforum.2022.4163 36416816 PMC9685483

[2] De Hert S. Burnout in healthcare workers: prevalence, impact and preventative strategies. Local Reg Anesth. 2020;13:171-183. doi:10.2147/LRA.S240564 33149664 PMC7604257

[3] Ben Simon E, Vallat R, Barnes CM, Walker MP. Sleep loss and the socio-emotional brain. Trends Cogn Sci. 2020;24(6):435-450. doi:10.1016/j.tics.2020.02.003 32299657

[4] Gurubhagavatula I, Barger LK, Barnes CM, . Guiding principles for determining work shift duration and addressing the effects of work shift duration on performance, safety, and health: guidance from the American Academy of Sleep Medicine and the Sleep Research Society. Sleep. 2021;44(11):zsab161. doi:10.1093/sleep/zsab161 34373924

[5] Stewart NH, Arora VM. The impact of sleep and circadian disorders on physician burnout. Chest. 2019;156(5):1022-1030. doi:10.1016/j.chest.2019.07.008 31352036 PMC6859241

[6] National Academies of Sciences, Engineering, and Medicine; National Academy of Medicine; Committee on Systems Approaches to Improve Patient Care by Supporting Clinician Well-Being. Taking Action Against Clinician Burnout: A Systems Approach to Professional Well-Being. National Academies Press; 2019. 31940160

