# Supplementary material for: Sleep Disturbance and Burnout in Emergency Department Health Care Workers
Source: JAMA Netw Open. 2023 Nov 3;6(11):e2341910. doi: 10.1001/jamanetworkopen.2023.41910 (PMC10625040; doi:10.1001/jamanetworkopen.2023.41910)
Supplement: Supplement 1. — eAppendix. Supplemental Methods eReferences [file jamanetwopen-e2341910-s001.pdf]

Supplemental Online Content

Shechter A, Firew T, Miranda M, et al. Sleep disturbance and burnout in emergency department health care workers. *JAMA Netw Open*. 2023;6(11):e2341910.
doi:10.1001/jamanetworkopen.2023.41910

eAppendix. Supplemental Methods

eReferences

This supplemental material has been provided by the authors to give readers additional information about their work.

© 2023 Shechter A et al. *JAMA Network Open.*

eAppendix. Supplemental Methods

Participants

Participants included healthcare workers (HCWs) from 4 emergency departments within a large academic medical center in New York City. Participants were recruited both in-person (during non-research related emergency department meetings and staff/team “huddles”) as well as via department-wide email list serves. Participants were eligible if they were over 18 years old and worked full time in the emergency department.

Outcomes

Participants completed a single online-based questionnaire via REDCap electronic survey. The survey included items on participant demographics (age, sex, and race/ethnicity) and a psychological test battery (e.g., depression, anxiety, stress). Demographics including race and ethnicity were classified by participant self-report.

Global sleep quality was assessed with the Pittsburgh Sleep Quality Index (PSQI).[1] The PSQI contains 19 items assessing sleep across 7 domains and generates a total global score ranging from 0 to 21 with higher scores indicating worse overall sleep quality. A PSQI score cutoff of >5 is used to indicate poor sleep quality.[1]

Insomnia symptoms were assessed with the Insomnia Severity Index (ISI).[2] The ISI contains 7 items and generates a total score of 0 to 28, with higher scores indicating worse severity insomnia symptoms. Scores 0-7 indicate no clinically significant insomnia; 8-14, subthreshold/mild insomnia; 15-21, clinical insomnia (moderate severity); 22-28, clinical insomnia (severe).[2]

Depression symptoms were assessed with the Patient Health Questionnaire-8 (PHQ-8).[3] Total scores range from 0 to 24, with scores in the range of 0 to 4 representing no significant depressive symptoms; 5 to 9, mild depressive symptoms; 10 to 14, moderate symptoms; 15 to 19, moderately severe symptoms; 20-24, severe symptoms.[4,5]

Burnout symptoms were assessed with the Abbreviated Maslach Burnout Inventory-9 (MBI-9).[6] The MBI-9 assessed the 3 burnout dimensions including emotional exhaustion (EE; feelings of being emotionally overextended and exhausted by one's work), depersonalization (DP; unfeeling and impersonal response towards patients), and reduced personal accomplishment (PA; feelings of competence and successful achievement in one's work). Scores on each component range from 0 to 18 with higher scores indicating more frequent symptoms for EE and DP, and lower scores indicating more frequent reduced PA. Cutoff scores of >9 indicated high EE, >6 high DP and <9 low PA.[7,8] As per recommendations, outcomes were analyzed as continuous scores on each domain, and also separately, categorical results using cutoffs for each domain.[9]

Analyses

Analyses were performed at the group level to account for the influence of organizational unit culture, a known contributory factor that impacts workforce and patient outcomes.[10]

Sample size

A post-hoc power analysis (G*Power) was conducted to determine the power to detect associations between sleep and burnout variables. The statistically significant positive Pearson correlations observed for the relationship between PSQI scores and depersonalization and

© 2023 Shechter A et al. *JAMA Network Open.*

emotional exhaustion ranged from 0.23 to 0.30, averaging 0.265. With a sample size of n=126, an observed correlation of ~0.265 and α = 0.05, the power of the sample is 0.86.

Safety measures

As part of the plan for protecting human subjects in the grant application and in the IRB, we outlined a safety protocol for use if participants indicated any suicidal ideations while completing study related assessments. If a participant indicated suicidal ideation, research staff would complete a 'Potential Suicidality form' and inform the study investigators who are physicians and psychologists. Based on the participant's presentation and history, a determination is made as to whether the participant is safe to participate in the study, whether the participant's primary care physician should be notified, and/or whether a formal consult and/or immediate referral for psychiatric care is necessary. None of the participants in the study indicated suicidal ideation.

eReferences

1. Buysse DJ, Reynolds III CF, Monk TH, Berman SR, Kupfer DJ. The Pittsburgh Sleep Quality Index: a new instrument for psychiatric practice and research. *Psychiatry research.* 1989;28(2):193-213.
2. Bastien CH, Vallières A, Morin CM. Validation of the Insomnia Severity Index as an outcome measure for insomnia research. *Sleep medicine.* 2001;2(4):297-307.
3. Kroenke K, Strine TW, Spitzer RL, Williams JB, Berry JT, Mokdad AH. The PHQ-8 as a measure of current depression in the general population. *Journal of affective disorders.* 2009;114(1-3):163-173.
4. Kroenke K, Spitzer RL. The PHQ-9: a new depression diagnostic and severity measure. In. Vol 32: Slack Incorporated Thorofare, NJ; 2002:509-515.
5. Strine TW, Mokdad AH, Balluz LS, et al. Depression and anxiety in the United States: findings from the 2006 behavioral risk factor surveillance system. *Psychiatric services.* 2008;59(12):1383-1390.
6. McManus I, Winder B, Gordon D. The causal links between stress and burnout in a longitudinal study of UK doctors. *The Lancet.* 2002;359(9323):2089-2090.
7. Riley MR, Mohr DC, Waddimba AC. The reliability and validity of three-item screening measures for burnout: Evidence from group-employed health care practitioners in upstate New York. *Stress and Health.* 2018;34(1):187-193.
8. McLoughlin C, Casey S, Feeney A, Weir D, Abdalla AA, Barrett E. Burnout, work satisfaction, and well-being among non-consultant psychiatrists in Ireland. *Academic Psychiatry.* 2021;45:322-328.
9. Dyrbye LN, West CP, Shanafelt TD. Defining burnout as a dichotomous variable. *Journal of general internal medicine.* 2009;24:440-440.
10. Jun J, Kovner CT, Dickson VV, Stimpfel AW, Rosenfeld P. Does unit culture matter? The association between unit culture and the use of evidence-based practice among hospital nurses. *Applied nursing research: ANR.* 2020;53:151251.

© 2023 Shechter A et al. *JAMA Network Open.*
