# Supplementary material for: Sleep Disturbance and Burnout in Emergency Department Health Care Workers
Source: JAMA Netw Open. 2023 Nov 3;6(11):e2341910. doi: 10.1001/jamanetworkopen.2023.41910 (PMC10625040; doi:10.1001/jamanetworkopen.2023.41910)
Supplement: Supplement 2. — Data Sharing Statement [file jamanetwopen-e2341910-s002.pdf]

Data Sharing Statement

Shechter. Sleep Disturbance and Burnout in Emergency Department Health Care Workers. *JAMA Netw Open*. Published November 07, 2023. doi:10.1001/jamanetworkopen.2023.41910

Data
Data available: Yes
Data types: Deidentified participant data
How to access data: Data can be made available upon request
When available: With publication

Supporting Documents
Document types: None

Additional Information
Who can access the data: Data will be made available to those who request and whose proposed use of the data has been approved
Types of analyses: analyses that have been approved
Mechanisms of data availability: will be made available after approval with a signed data access agreement
